# Conversion to Complete, Parenchyma-Sparing Resection after Prolonged Denosumab for Bilateral Multifocal Pulmonary Metastases from Giant Cell Tumor of Bone: A Case Report

**DOI:** 10.5761/atcs.cr.25-00214

**Published:** 2026-01-23

**Authors:** Seiji Omura, Aya Sasaki, Ukei Anazawa, Keisuke Eguchi

**Affiliations:** 1 Department of General Thoracic Surgery, Tokyo Dental College, Ichikawa General Hospital, Ichikawa, Chiba, Japan; 2 Department of Pathology and Laboratory Medicine, Tokyo Dental College, Ichikawa General Hospital, Ichikawa, Chiba, Japan; 3 Department of Orthopedic Surgery, Tokyo Dental College, Ichikawa General Hospital, Ichikawa, Chiba, Japan

**Keywords:** giant cell tumor of bone, pulmonary metastases, denosumab, video-assisted thoracoscopic surgery (VATS), metastasectomy

## Abstract

Giant cell tumor of bone (GCTB) rarely metastasizes, but pulmonary lesions pose therapeutic challenges. We report a woman in her 30s who developed multiple bilateral lung nodules 3.5 years after distal ulna GCTB resection and local recurrences. Denosumab 120 mg every 4 weeks was given for 2.5 years, producing shrinkage, calcification, and stability. Staged, palpation-guided thoracoscopic wedge resections (8 left, 5 right) achieved complete macroscopic clearance with negative margins. Histology showed spindle-cell proliferation with woven bone and depletion of giant cells; H3.3 G34W immunostaining confirmed metastatic GCTB. She remains recurrence-free 7 years and 5 months after metastasectomy. Denosumab displayed site-specific surgical implications—unfavorable at the primary bone site due to peritumoral sclerosis, yet advantageous in the lung where it clarifies margins and enables parenchyma-sparing R0 resection. A surgery-forward strategy that uses time-limited denosumab as a bridge to meticulous thoracoscopic metastasectomy may secure durable control in multifocal pulmonary GCTB.

## Introduction

Giant cell tumor of bone (GCTB) is a rare, locally aggressive primary bone neoplasm that typically arises in the meta-epiphyseal region of long bones in young adults.^1,2)^ Although classified as an intermediate tumor, GCTB exhibits a distinctive capacity for local recurrence and can metastasize to the lung in approximately 1%–9% of cases.^1–4)^ The discovery of recurrent H3F3A mutations, most commonly p.G34W, in neoplastic stromal cells has refined the diagnostic definition of GCTB and enables mutation-specific immunohistochemical confirmation in treatment-modified specimens.^5–7)^

Denosumab, a monoclonal antibody against receptor activator of nuclear factor-κB ligand, has broadened systemic options for unresectable or multifocal GCTB. By inhibiting osteoclastogenesis, it induces tumor shrinkage, osteoid formation, and radiographic sclerosis, sometimes facilitating surgical down-staging.^8–10)^ However, prolonged preoperative administration at the primary site can induce dense peripheral sclerosis and new bone formation that obscure viable tumor boundaries, potentially leading to residual disease after marginal or intralesional resection and increasing local-recurrence risk—a well-recognized negative surgical implication of denosumab in skeletal GCTB.^11,12)^

In contrast, its influence on the surgical management of pulmonary metastases remains poorly defined. Because lung nodules arise within aerated parenchyma, denosumab-induced calcification and peripheral delineation may, paradoxically, improve resectability by clarifying tumor margins and enabling precise, parenchyma-sparing wedge resections; whether these morphologic effects translate into favorable surgical outcomes has not been well documented.^13–15)^

Here, we describe a young woman with bilateral, multifocal pulmonary metastases from conventional GCTB who received a 2.5-year course of denosumab followed by staged, palpation-guided thoracoscopic wedge resections achieving complete clearance. This case illustrates the site-specific surgical implications of denosumab—unfavorable at the primary bone site but advantageous in the lung—and supports a surgery-forward approach integrating time-limited systemic therapy with meticulous, parenchyma-preserving metastasectomy.

## Case Report

**Fig. 1:**
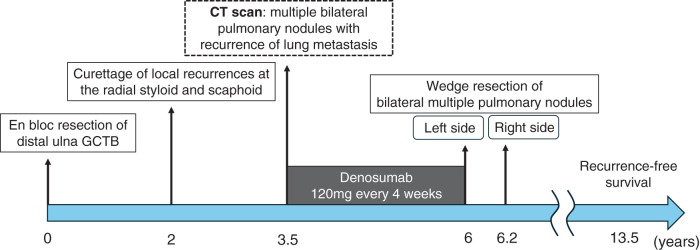
Clinical course of the patient. Timeline illustrating the clinical course of a woman in her 30s with GCTB. She initially underwent en bloc resection of distal ulna GCTB, followed by curettage of local recurrences at the radial styloid and scaphoid 2 years later. At 3.5 years after the index surgery, chest CT revealed multiple bilateral pulmonary nodules. Denosumab (120 mg every 4 weeks) was administered for approximately 2.5 years, achieving shrinkage and stabilization of the lesions. Staged video-assisted thoracoscopic wedge resections of bilateral multiple pulmonary nodules were subsequently performed (left then right, 7 weeks apart), achieving complete macroscopic clearance. The patient remains recurrence-free 7 years and 5 months after metastasectomy. GCTB: giant cell tumor of bone; CT: computed tomography

**Fig. 2:**
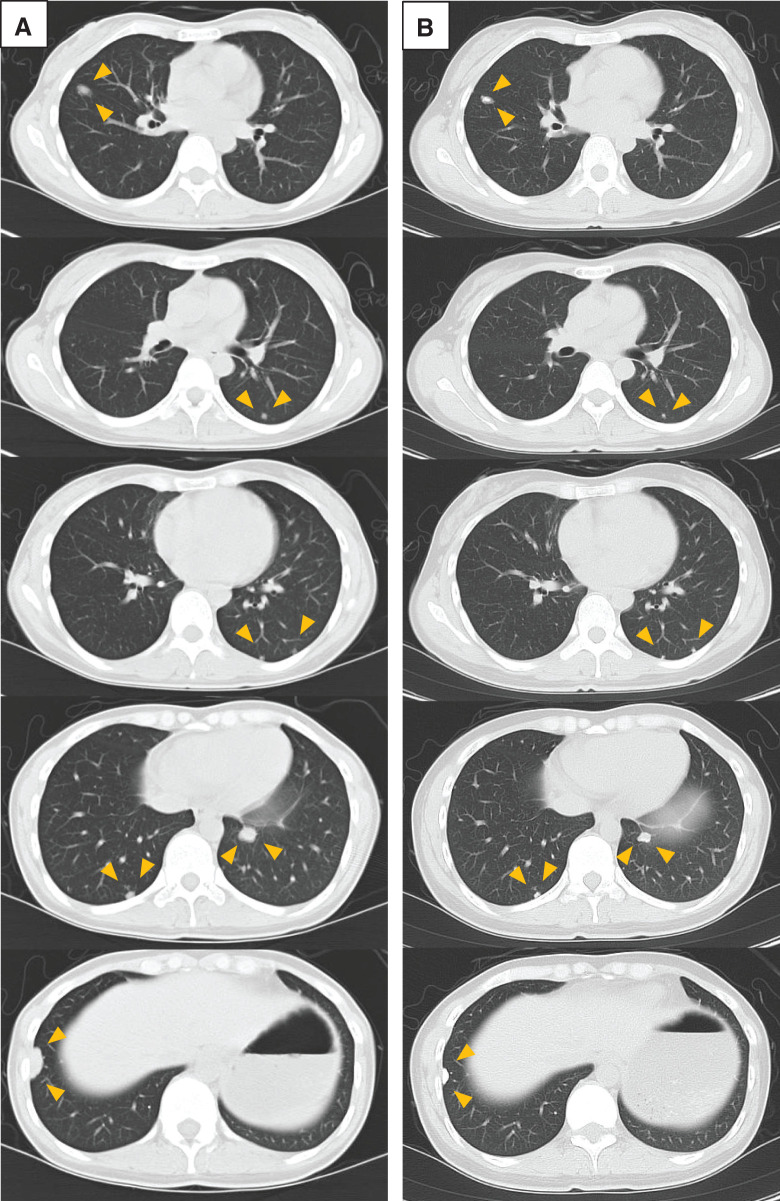
Serial chest CT findings before and after denosumab therapy. (**A**) Chest CT obtained before initiation of denosumab showing multiple, predominantly peripheral bilateral pulmonary nodules consistent with metastatic GCTB. (**B**) Chest CT after approximately 2.5 years of denosumab treatment demonstrating shrinkage, calcification, and stabilization of the nodules prior to metastasectomy. CT: computed tomography; GCTB: giant cell tumor of bone

A woman in her 30s underwent en bloc resection of a distal ulna GCTB at our institution. Two years later, local recurrences developed at the radial styloid and scaphoid, each treated by curettage and confirmed as conventional (non-malignant) GCTB (**Fig. 1**). At 3.5 years after the index operation, surveillance chest computed tomography (CT) revealed multiple, predominantly peripheral bilateral pulmonary nodules in an otherwise asymptomatic patient (**Fig. 2A**).

Denosumab was initiated and continued for approximately 2.5 years, resulting in sustained shrinkage, calcification, and stabilization of the lesions (**Fig. 2B**). Six years after diagnosis, with the disease limited and technically resectable, definitive surgery was undertaken.

**Fig. 3:**
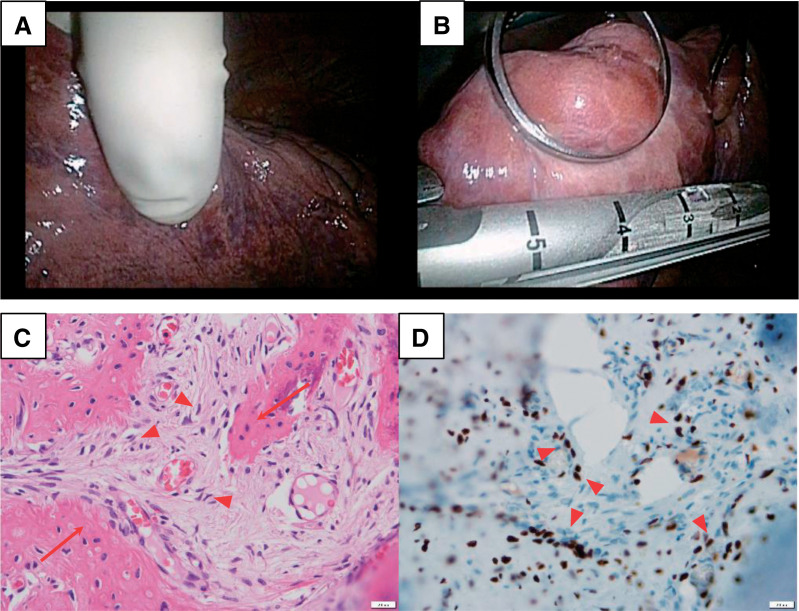
Intraoperative and histopathologic findings of pulmonary metastases from GCTB. (**A**, **B**) Intraoperative photographs during video-assisted thoracoscopic surgery demonstrating meticulous inspection and bimanual palpation of all lobes to identify small, denosumab-treated peripheral nodules, followed by parenchyma-sparing wedge resection with deliberate margin acquisition. (**C**) Histologic section showing spindle-cell proliferation with conspicuous woven bone formation and depletion of osteoclast-like multinucleated giant cells, consistent with denosumab-related treatment effect (hematoxylin and eosin stain). The arrows indicate woven bone trabeculae; the arrowheads indicate spindle-shaped mononuclear stromal cells. (**D**) Immunohistochemistry for H3.3 G34W demonstrating nuclear positivity in mononuclear stromal cells, confirming metastatic giant cell tumor of bone. The arrowheads indicate H3.3 G34W-positive nuclei. GCTB: giant cell tumor of bone

Under general anesthesia with 1-lung ventilation, left-sided thoracoscopic surgery was performed using two 12-mm ports and a 40-mm mini-thoracotomy. Thorough inspection combined with systematic bimanual palpation identified small, ossified peripheral nodules invisible on the pleural surface (**Figs. 3A** and **3B**). Eight lesions were removed by wedge resections with deliberate margin acquisition. Seven weeks later, a similar right-sided procedure yielded 5 additional resections. All stapled margins were negative; recovery was uneventful, and denosumab was discontinued postoperatively.

Histopathology showed spindle-cell proliferation with conspicuous woven bone formation and depletion of osteoclast-like multinucleated giant cells—consistent with denosumab effect (**Fig. 3C**). No cytologic atypia or necrosis was observed. Immunohistochemistry for H3.3 G34W demonstrated nuclear positivity in stromal cells, confirming metastatic GCTB (**Fig. 3D**). The patient remains alive and disease-free 7 years 5 months after metastasectomy.

## Discussion

This case highlights 3 key considerations in the thoracic management of metastatic GCTB.

First, intensified chest surveillance after local recurrence allows timely detection of pulmonary metastases, which often present as small, peripheral nodules suitable for limited resection. Chest CT surpasses radiography for identifying subcentimeter lesions and is particularly valuable during the first 3 years after recurrence, when metastatic events tend to cluster.^16)^ Notably, the risk of pulmonary dissemination is higher after local recurrence and with advanced Campanacci stage and specific anatomic sites, underscoring the need for risk-adapted follow-up.^3,4)^

Second, while observation may be reasonable for small, indolent nodules, progression is common—particularly for lesions exceeding several millimeters—and many patients ultimately require surgery.^3,4,15,17)^ Denosumab can stabilize or downsize multifocal disease and serve as a bridge to operability; however, its effects differ strikingly between primary and metastatic sites. At skeletal primaries, denosumab induces osteoid deposition and dense peripheral sclerosis within which viable tumor cells may persist. This remodeling blurs the tumor–host interface and complicates margin assessment, increasing local-recurrence risk after marginal or intralesional procedures.^11,12)^ In contrast, within lung parenchyma, treatment-related calcification and peripheral delineation can sharpen resection planes and facilitate parenchyma-sparing, margin-negative wedge resections. In our patient, all margins were histologically negative with no residual tumor identified, supporting that denosumab’s adverse surgical implications in bone do not extend to the pulmonary setting.^8–10,13)^ Denosumab thus shows a site-specific duality—potentially detrimental at the primary site yet beneficial for metastasectomy in the lung.

Third, surgical planning should reconcile oncologic radicality with functional preservation, particularly in young patients with bilateral disease. Contemporary thoracic principles advocate complete removal of all macroscopic disease with maximal parenchymal conservation and staged procedures for bilateral lesions; meticulous thoracoscopic inspection coupled with systematic palpation of every lobe remains essential because small, treatment-altered nodules may be inconspicuous on the pleural surface yet readily detectable by tactile assessment.^13,14)^ Employing this strategy, we achieved complete macroscopic clearance without morbidity and observed durable disease-free survival beyond 7 years, consistent with prior series showing excellent outcomes after complete metastasectomy in GCTB.^15,17)^

Histopathologic confirmation using H3.3 G34W immunohistochemistry was particularly valuable in this denosumab-modified context, enabling discrimination between therapy-induced stromal remodeling and viable GCTB and assisting in the exclusion of malignant transformation, which—though uncommon—carries a worse prognosis.^6,7,18)^

Taken together, our experience supports a pragmatic, surgery-forward pathway for bilateral, multifocal GCTB lung metastases:

1) CT-based intensified surveillance for early detection,^16)^ with attention to high-risk features;^3,4)^
2) goal-directed, time-limited denosumab to achieve disease stabilization and convert to operability while avoiding overtreatment;^8–12)^ and
3) staged, palpation-guided, parenchyma-sparing thoracoscopic metastasectomy with deliberate margin acquisition to ensure complete clearance, consistent with thoracic consensus.^13,14)^

## Conclusion

In this patient with bilateral, multifocal pulmonary metastases from conventional GCTB, prolonged denosumab therapy converted multifocal disease to a surgically manageable state. Subsequent staged, palpation-guided, parenchyma-sparing wedge resections achieved complete macroscopic clearance and long-term disease-free survival. Denosumab exhibited distinct site-specific surgical implications—unfavorable at the primary bone site due to peritumoral sclerosis that may harbor residual cells, yet favorable in the lung where peripheral delineation facilitated precise, margin-negative resection. These findings reinforce a surgery-forward strategy that combines risk-adapted CT surveillance, time-limited denosumab as a bridge to operability, and complete resection as the cornerstone of durable control in metastatic GCTB.

## Declarations

### Data Availability

The data that support the findings of this study are available from the corresponding author upon reasonable request.
